# Urinary Biomarkers Indicative of Apoptosis and Acute Kidney Injury in the Critically Ill

**DOI:** 10.1371/journal.pone.0149956

**Published:** 2016-02-26

**Authors:** Suvi T. Vaara, Päivi Lakkisto, Katariina Immonen, Ilkka Tikkanen, Tero Ala-Kokko, Ville Pettilä, FINNAKI Study Group

**Affiliations:** 1 Division of Intensive Care Medicine, Department of Anaesthesiology, Intensive Care and Pain Medicine, University of Helsinki and Helsinki University Hospital, Helsinki, Finland; 2 Minerva Institute for Medical Research, Helsinki, Finland; 3 Department of Clinical Chemistry and Hematology, University of Helsinki and Helsinki University Hospital, Helsinki, Finland; 4 Abdominal Center, Nephrology, University of Helsinki and Helsinki University Hospital, Helsinki, Finland; 5 Department of Anaesthesiology, University of Oulu and Division of Intensive Care Medicine, Oulu University Hospital, Medical Research Center Oulu, Oulu, Finland; 6 Department of Intensive Care Medicine, Bern University Hospital (Inselspital), University of Bern, Bern, Switzerland; Bambino Gesù Children's Hospital, ITALY

## Abstract

**Background:**

Apoptosis is a key mechanism involved in ischemic acute kidney injury (AKI), but its role in septic AKI is controversial. Biomarkers indicative of apoptosis could potentially detect developing AKI prior to its clinical diagnosis.

**Methods:**

As a part of the multicenter, observational FINNAKI study, we performed a pilot study among critically ill patients who developed AKI (n = 30) matched to critically ill patients without AKI (n = 30). We explored the urine and plasma levels of cytokeratin-18 neoepitope M30 (CK-18 M30), cell-free DNA, and heat shock protein 70 (HSP70) at intensive care unit (ICU) admission and 24h thereafter, before the clinical diagnosis of AKI defined by the Kidney Disease: Improving Global Outcomes -creatinine and urine output criteria. Furthermore, we performed a validation study in 197 consecutive patients in the FINNAKI cohort and analyzed the urine sample at ICU admission for CK-18 M30 levels.

**Results:**

In the pilot study, the urine or plasma levels of measured biomarkers at ICU admission, at 24h, or their maximum value did not differ significantly between AKI and non-AKI patients. Among 20 AKI patients without severe sepsis, the urine CK-18 M30 levels were significantly higher at 24h (median 116.0, IQR [32.3–233.0] U/L) than among those 20 patients who did not develop AKI (46.0 [0.0–54.0] U/L), *P* = 0.020. Neither urine cell-free DNA nor HSP70 levels significantly differed between AKI and non-AKI patients regardless of the presence of severe sepsis. In the validation study, urine CK-18 M30 level at ICU admission was not significantly higher among patients developing AKI compared to non-AKI patients regardless of the presence of severe sepsis or CKD.

**Conclusions:**

Our findings do not support that apoptosis detected with CK-18 M30 level would be useful in assessing the development of AKI in the critically ill. Urine HSP or cell-free DNA levels did not differ between AKI and non-AKI patients.

## Introduction

Acute kidney injury (AKI) is a common syndrome among the critically ill [1] that increases mortality and morbidity [2]. Currently, the diagnosis of AKI is made using functional criteria based on rise in plasma creatinine and/or decreased urine output [3]. Neither of these is ideal, and, novel, sensitive, and early biomarkers to detect kidney injury are warranted [4]. Discovery of novel and sensitive biomarkers able to detect AKI prior its clinical diagnosis could provide not only new insights into the multifactorial pathophysiology of AKI involving ischemic, toxic, and inflammatory insults to the kidneys [3], but also allow development of new preventive strategies. Apoptosis of epithelial cells in proximal and distal tubules is a proposed mechanism involved both in ischemic and nephrotoxic AKI [5], whereas evidence showing no trace of apoptosis in septic AKI is accumulating [6, 7].

Caspases are proteins that mediate cell apoptosis once it has been induced by other signals. Cytokeratin-18 is an intermediate filament protein abundant in simple epithelial cells [8] that is cleaved by caspases during apoptosis, and the caspase-cleaved fragment M30 of cytokeratin-18 can be measured [9, 10]. In healthy individuals, apoptosis is the primary mechanism leading to the detection of cell-free DNA in plasma [11], and cell-free DNA detected in urine has been shown to originate from the injured kidneys [12]. Heat shock protein 70 (HSP70) is a chaperon protein that protects cells from various damaging stress (e.g. ischemia) and also a powerful inhibitor of apoptosis [13]. Induction of HSP70 has been shown to protect from cell damage and reduce the rate of apoptosis by modulating caspase activity [13]. Moreover, HSP70 has been shown to be an early marker of ischemia-reperfusion injury associated AKI in rats [14]. Elevated HSP70 levels in urine have been detected in a small study of critically ill patients before the clinical diagnosis of AKI [14, 15]. None of these three markers have been studied in a reasonable-sized cohort of critically ill patients with AKI.

We aimed to study the role of these three apoptosis-related biomarkers in detecting patients developing AKI. As a part of the multicenter FINNAKI cohort study, we performed a pilot study among critically ill patients who developed AKI matched to critically ill patients without AKI and explored the urine and plasma levels of cytokeratin-18 neoepitope M30 (CK-18 M30), cell-free DNA, and HSP70 at ICU admission and 24h thereafter, before the clinical diagnosis of AKI and according to the presence of septic AKI. Based on the results of this pilot study, we analyzed CK-18 M30 urine levels at ICU admission in a larger validation cohort.

## Materials and Methods

This was a subanalysis of the prospective, multicenter Finnish Acute Kidney Injury (FINNAKI) Study conducted between 1 Sep, 2011 and 1 Feb, 2012 in 17 Finnish intensive care units (ICUs). The Ethics Committee of the Department of Surgery, Helsinki and Uusimaa Hospital District approved the study protocol and the use of deferred consent with a written, informed consent obtained as soon as possible. Each participant or his/her proxy gave written consent. The study was conducted according to the Declaration of Helsinki.

Patients with an emergency admission or with a post-surgical admission with an expected duration >24 h were eligible for the FINNAKI study. Exclusion criteria were: 1) presence of end-stage renal disease and requirement for maintenance dialysis, 2) previous use of renal replacement therapy (RRT) while enrolled in the study, 3) organ donors, 4) patients not permanently living in Finland, 5) intermediate care patients, 6) transferred patients from other study ICUs if the study data collection period had been fulfilled, 7) absence of consent. Only patients with available samples were included in the current analysis.

### Pilot study

For the pilot study, we further excluded the following: 1) patients with pre-existing chronic kidney disease (CKD) (GFR <60ml/min/1.73m^2^), 2) patients with AKI diagnosed within first 24h from ICU admission. Of eligible patients, we randomly selected 30 patients diagnosed with AKI and 30 patients without AKI enrolled in the FINNAKI study between 1 Dec, 2011 and 1 Feb, 2012. This pilot cohort was a convenience sample because previous reliable mean values and distributions of each laboratory measurement were not available for sample size calculations. For comparison of two groups 10 patients per group is considered as the minimum. We aimed to reduce the heterogeneity in demographic data by using a predetermined case-control matching strategy to increase the power of the study. We matched the non-AKI patients to AKI patients before analyses.

We entailed the group of AKI patients to include 10 patients with severe sepsis diagnosed within 24h of ICU admission and as many patients with AKI stage 2 or 3 as available. AKI patients were matched to non-AKI controls according to 1) age (caliper width 5 years), 2) sex, 3) presence of severe sepsis, and 4) the Simplified Acute Physiology II score (SAPS II) without renal and age components (caliper width 5 points). Biomarker levels were studied first in the whole cohort, and then in the subcohort of non-septic patients. The primary end-point was development of new AKI after 24h of ICU treatment.

### Validation study

According to the results of the pilot study, we aimed to show a difference in the urine CK-18 M30 levels of corresponding magnitude as observed in the pilot among patients without sepsis. With a two-sided alpha of 0.05 and power of 90%, 46 AKI cases would be needed. In order to gain maximum external validity, we analyzed a consecutive sample from the beginning of the cohort including also septic patients and patients with CKD. Therefore, for the validation study, we analyzed the urine samples at ICU admission of 197 consecutive patients enrolled in the FINNAKI study who were not included in the pilot study. Of these consecutive potentially eligible patients, 16 (8 of these developed AKI during the study period) did not have urine sample available and were excluded. The validation cohort included 79 AKI patients (48 with non-septic and 31 with septic AKI).

### Definition of AKI and severe sepsis

We defined AKI with the Kidney Disease: Improving Global Outcomes (KDIGO) criteria [3] using both daily creatinine measurements and hourly urine output. As a baseline creatinine we used the latest creatinine obtained within last year but not within last week. If not available, we calculated it using the MDRD equation and assuming a GFR of 75ml/min/1.73m^2^ as recommended [16]. Attending intensivists screened the patients on admission and thereafter daily for presence of severe sepsis according to the American College of Chest Physicians/Society of Critical Care Medicine (ACCP/SCCM) definition [17].

### Data collection

Routine patient characteristics, physiological and laboratory data, and disease severity scores were collected from the ICU data management system via the Finnish Intensive Care Consortium database. Supplemental data on pre-existing chronic illnesses, medication, and risk factors for AKI were recorded using case report forms. The data collection period for clinical data was the first five days in the ICU. We calculated the sum of various nephrotoxic substances (maximum of 6) a patient had received prior to ICU admission, and included the following: radiocontrast agent, aminoglycan or peptidoglycan antibiotics, angiotensin converting enzyme inhibitors or angiotensin receptor blockers, non-steroidal anti-inflammatory drugs, diuretics, or hydroxyethyl starch.

### Laboratory samples

Blood and urine samples were immediately collected once patient was admitted to ICU (H0 sample) and 24 h later (H24 sample). Urine samples were centrifuged and frozen. Blood samples were collected in EDTA tubes and centrifuged. Plasma samples were transferred in acid-handled plastic tubes and frozen. Urine and plasma samples were kept in -80 degrees until analyzed in June 2013 in the pilot study and in January 2015 in the validation study.

### Determination of plasma and urine cell-free DNA

Plasma and urine cell-free DNA was extracted as described previously [18]. Both plasma and urine samples were centrifuged at 16000g for 10 minutes before DNA extraction to remove any residual cells [19]. DNA extraction was performed using the QIAamp DNA Blood Mini Kit (Qiagen, Hilden, Germany) according to the “blood and body fluid protocol” as recommended by the manufacturer.

Cell-free DNA was measured from duplicate samples using TaqMan Gene Expression assay for human β-globin gene (Hs00758889_s1, Life Technologies, USA) and TaqMan gene expression master mix (Life Technologies). The assay is suitable for measuring genomic DNA as both primers and probe lie within a single exon. The amplicon length of the PCR product is 95 bp. The PCR reactions were performed using Roche LightCycler 480 real-time PCR system (Roche, Mannheim, Germany). A 10-fold serial dilution of human genomic DNA (Roche) was used as a standard curve. Results are expressed as genome equivalents (GE) per milliliter (mL). One GE equals 6.6 pikogram of DNA.

### Determination of plasma and urine CK-18 M30 and HSP70

Plasma and urine concentrations of CK-18 M30 and HSP70 were measured using M30-Apoptosense® ELISA kit (Peviva AB, Bromma, Sweden) and HSP70 high-sensitivity ELISA kit (Enzo Life Sciences, USA), respectively. Assays were performed according to manufacturers’ instructions. The detection limit of CK-18 M30 ELISA assay is 25 U/l and the intra- and interassay CV <10%, as reported by manufacturer. For HSP70 ELISA assay, the detection limit is 0.09 mg/ml, the intra-assay CV 3.9% to 11.4% and interassay CV 12.8% to 19.1%, as reported by the manufacturer.

### Statistical analyses

We analyzed the biomarker levels measured at H0, H24, and the maximum value of these two measurements as three separate variables. We studied the normality of continuous variables with the Kolmogorov-Smirnov test. As they were not normally distributed, we report them with medians and interquartile ranges (IQR; 25-75^th^ percentiles) and categorical variables with count and percentage. We compared continuous data with Mann-Whitney U-test and categorical data with Fisher’s exact test. We calculated the difference between medians with 95% confidence interval using the Hodges-Lehmann approach. Correlations between these non-normally distributed variables were assessed with Spearman’s rho. We considered a two-sided P <0.05 significant and did not correct for multiple comparisons. We conducted analyses with SPSS Statistics 20.0 for Mac (IBM, Armonk, NY) with a supplemental algorithm “FUZZY” for case-control matching and PRISM 6.0 (GraphPad Software, La Jolla, CA).

## Results

### Pilot study

#### AKI versus non-AKI patients

Between AKI patients and their matched non-AKI controls, no significant differences in patient characteristics and non-renal disease severity remained after matching (S1 Table). Of the 30 patients with AKI, 18 (60.0%) had stage 1 AKI, 6 (20.0%) stage 2 AKI, and 6 (20.0%) stage 3 AKI. All stage 3 patients received renal replacement therapy (RRT). The median [IQR] time from ICU admission to AKI diagnosis was 3 [2.0–3.3] days.

The urine levels of measured biomarkers at H0, H24, or the maximum value of these two did not differ significantly between AKI and non-AKI patients (Table 1). No correlation between the severity of AKI and urine levels at H24 of any measured biomarker were detected. No significant differences were observed in the plasma levels of the biomarkers between all AKI and non-AKI patients at any time-point (S2 Table).

**Table 1:** Urine biomarker levels in all patients with acute kidney injury (AKI) and their matched non-AKI controls in the pilot study.

	AKI (n = 30)	No AKI (n = 30)	P-value	Difference in group median with 95% CI
**Caspase-cleaved cytokeratin-18 epitope M30 (U/L)**				
-0h	48.0 [3.8–102.3]	43.0 [15.0–65.0]	0.577	3.0 [-19.0–35.0]
-24h	63.0 [7.5–124.8]	46.0 [0.0–73.3]	0.173	15.0 [-1.0–63.0]
-highest	116.0 [63.0–302.0]	54.0 [11.5–94.3]	0.182	28.5 [-9.0–69.0]
**Cell-free DNA (GE/mL)**				
-0h	33.5 [0.0–386.5]	67.5 [0.0–564.0]	0.671	0.0 [-130.0–19.0]
-24h	0.0 [0.0–406.8]	19.0 [0.0–218.3]	0.994	0.0 [-19.0–9.0]
-highest	430.0 [11.5–983.8]	73.5 [0.0–733.8]	0.905	0.0 [-206.0–217.0]
**HSP (ng/mL)**				
-0h	1.13 [0.47-1-43]	1.13 [0.40–1.95]	0.667	-0.07 [-0.66–0.33]
-24h	1.17 [0.00–2.23]	1.00 [0.40–1.85]	0.911	0.0 [-0.47–0.73]
-highest	1.30 [0.73–2.38]	1.40 [1.05–1.98]	0.865	0.0 [-0.54–0.87]

Data expressed as median [IQR]. CI; confidence interval

#### Non-septic patients with AKI versus without AKI

**Fig 1:**
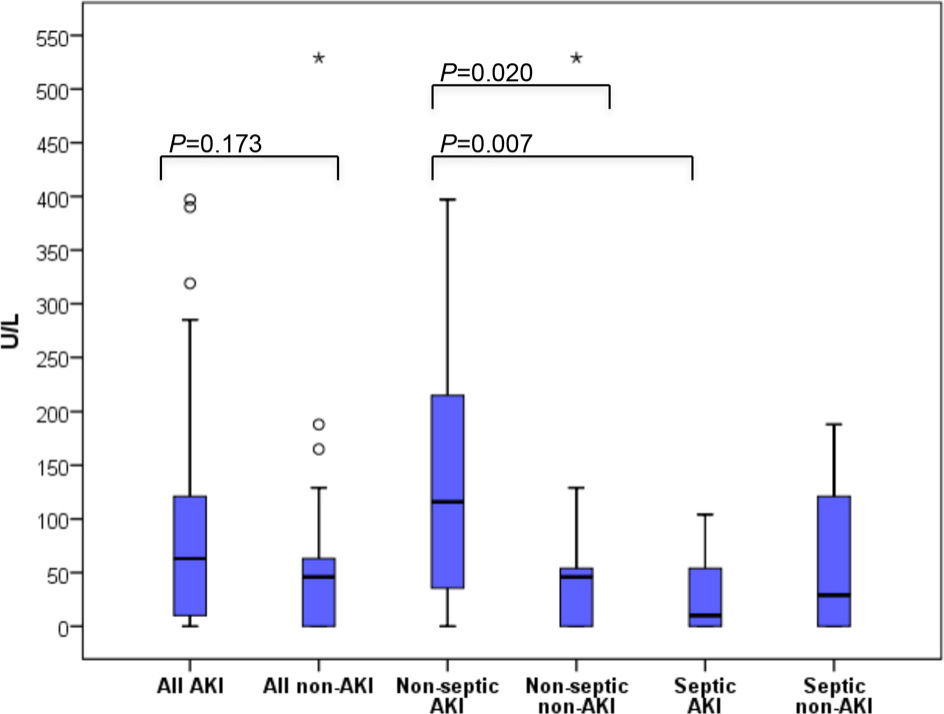
Box-plots presenting urine caspase-cleaved cytokeratin epitope M30 (U/l) levels measured 24 hours from ICU admission in the pilot study. AKI; acute kidney injury.

Among patients without severe sepsis, AKI patients (n = 20) had significantly higher urine CK-18 M30 levels at H24 compared to those without AKI (n = 20) (Fig 1). The median [IQR] time from H24 to the diagnosis of AKI was 21.4 [5.2–41.4] hours. The urine levels of HSP or cell-free DNA did not differ significantly at any time-point (Table 2). Among non-septic AKI patients, urine CK-18 M30 did not show significant correlation at H24 with cell-free DNA (Spearman’s rho -0.223, *P* = 0.345) or HSP70 (Spearman’s rho 0.108, *P* = 0.650) or the severity of AKI (Spearman’s rho 0.194, *P* = 0.413). The plasma levels of the measured markers did not differ between non-septic patients with or without AKI (S3 Table).

**Table 2:** Urine biomarker levels in non-septic patients with and without acute kidney injury (AKI).

	AKI (n = 20)	No AKI (n = 20)	P-value	Difference in group median with 95% CI
**Caspase-cleaved cytokeratin-18 epitope M30 (U/L)**				
-0h	61.5 [31.3–107.0]	39.0 [6.5–61.0]	0.157	17.0 [-5.0–50.0]
-24h	116.0 [32.3–233.0]	46.0 [0.0–54.0]	0.020	66.0 [9.0–121.0]
-highest	116.0 [63.0–302.0]	54.0 [11.5–94.3]	0.010	62.0 [9.0–125.0]
**Cell-free DNA (GE/mL)**				
-0h	44.0 [0.0–453.5]	38.0 [0.0–733.8]	0.904	0.0 [-58.0–188.0]
-24h	9.5 [0.0–570.3]	0.0 [0.0–48.8]	0.211	0.0 [0.0–300.0]
-highest	430.0 [11.5–983.8]	73.5 [0.0–733.8]	0.253	74.0 [-50.0–489.0]
**HSP (ng/mL)**				
-0h	1.13 [0.73–1.50]	1.07 [0.40–1.80]	0.698	0.07 [-0.53–0.73]
-24h	0.67 [0.00–2.04]	1.23 [0.42–1.73]	0.529	-0.23 [-0.80–0.47]
-highest	1.30 [0.73–2.38]	1.40 [1.05–1.98]	0.779	-0.07 [-0.73–0.67]

Data expressed as median [IQR]. CI; confidence interval

### Validation study

Of the 197 included patients, 79 (40.1%) had AKI. Of these, 31 (39.2%) developed stage 1 AKI, 17 (21.5%) stage 2 AKI, and 31 (39.2%) stage 3 AKI. Altogether 25 (12.7%) of the 197 patients received RRT. The 79 AKI patients included 24 (30.4%) patients with stage 2 or 3 according to the urine output criteria. The diagnosis of AKI was made within the first 12h in the ICU in 43 (54.4%), within 12h to 24h of ICU treatment in 25 (31.6%), on day 2 in 5 (6.3%), and 6 (7.6%) patients were diagnosed thereafter.

#### All AKI patients versus non-AKI patients

Patients with AKI had more frequently pre-existing CKD (17.7%) than those who did not develop AKI (3.4%), P<0.001, and AKI patients had received more nephrotoxic substances before ICU admission (S4 Table). The median [IQR] urine CK-18 M30 level among AKI patients was 57.3 [34.1–103.1] and among non-AKI patients 59.6 [37.5–106.0] (U/L), p = 0.655. The CK-18 M30 level among patients with AKI diagnosed over 24h from ICU admission was 52.9 [38.6–110.8] U/L. Among AKI patients without CKD (n = 65), the median [IQR] urine CK-18 M30 level was 57.3 [32.0–107.0] and of non-AKI patients (n = 113) 58.3 [37.4–107.2] U/l, p = 0.701. In the 24 patients with stage 2 or 3 oliguric AKI, the median [IQR] urine M30 level at ICU admission was 76.1 [27.6–199.2] U/L, not different from the non-AKI patients (p = 0.681). No difference was detected in the M30 levels according to the severity of AKI.

#### Non-septic AKI versus non-septic non-AKI patients

**Fig 2:**
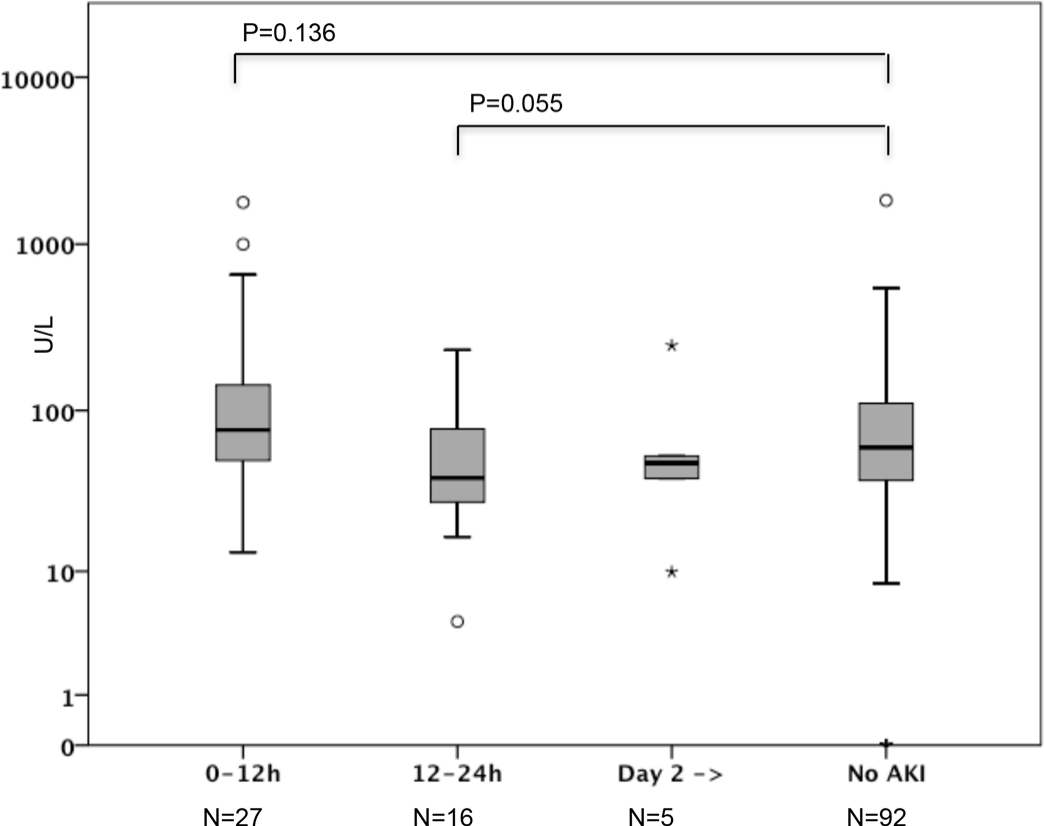
Urine CK-18 M30 levels measured at ICU admission among non-septic patients included in the validation cohort according to the time of acute kidney injury (AKI) diagnosis.

Of these 140 patients, 48 (34.2%) had AKI and 18 (12.9%) received RRT. AKI and non-AKI patients did not differ regarding severity of illness according to the SAPS II score without renal and age components (Table 3). The first AKI diagnosis was made in 27 (56.3%) within first 12h, in 16 (33.3%) within 12 to 24h of ICU admission, and in 5 (10.4%) on day 2 or 3. Among these non-septic patients, the median [IQR] urine CK-18 M30 concentration in AKI patients was 61.5 [34.2–102.2] U/L and in non-AKI patients 59.6 [37.5–111.2] U/L, p = 0.899. No significant differences existed when AKI patients were compared to non-AKI patients grouped according to the time of AKI diagnosis (Fig 2). Patients with AKI diagnosed within 12h of ICU admission (n = 27) had higher median [IQR] CK-18 M30 levels 76.1 [49.2] U/L compared to those with AKI diagnosed later (n = 21) 44.0 [27.6–77.3] U/L, p = 0.015. When patients with pre-existing CKD were excluded, median [IQR] urine CK-18 M30 level in AKI patients (n = 40) was 61.5 [29.7–102.2] U/L and in non-AKI patients (n = 88) 59.6 [37.3–111.2] U/L, p = 0.884.

**Table 3:** Characteristics of non-septic patients with and without acute kidney injury (AKI) in the validation study.

	AKI (n = 48)	Non-AKI (n = 92)	P-value
Age	65.0 [48.8–73.5]	62.5 [49.0–73.0]	0.881
Male sex	36/48 (75.0%)	63/92 (68.5%)	0.443
Hypertension	25/47 (53.2%)	45/91 (49.5%)	0.722
Diabetes mellitus	8/48 (16.7%)	16/72 (17.4%)	>0.999
Chronic kidney disease	8/48 (16.7%)	4/92 (4.3%)	0.023
Number of received nephrotoxic agents prior to ICU admission^a^	1.5 [1.0–3.0]	1.0 [0–2.0]	0.007
Operative admission	18/48 (37.5%)	32/92 (34.8%)	0.853
Emergency admission	37/43 (86.0%)	86/91 (94.5%)	0.174
SAPS II score	38.5 [29.0–50.8]	32.0 [25.0–41.8]	0.027
Non-renal non-age SAPS II score	18.5 [14.0–25.0]	20.5 [13.0–29.3]	0.627
SOFA score, maximum	9 [7–12]	6 [3–9]	<0.001
Ventilatory treatment	34/48 (70.8%)	55/92 (59.8%)	0.267
Vasoactives on day 1	35/48 (72.9%)	37/92 (40.2%)	<0.001
Urine output, first 24h	1242 [675–2155]	2740 [1763–3963]	<0.001
Length of ICU stay	3.8 [1.9–7.2]	2.1 [1.0–4.0]	0.002
Dead by day 90	12/48 (25.0%)	18/92 (19.6%)	0.517

Data expressed as median [IQR] or number/total number (%). ICU; intensive care unit, SAPS; Simplified Acute Physiology Score, SOFA; Sequential Organ Dysfunction Assessment

^a^ Included (maximum of 6): radiocontrast agent, aminoglycan or peptidoglycan antibiotics, angiotensin converting enzyme inhibitors or angiotensin receptor blockers, non-steroidal anti-inflammatory drugs, diuretics or hydroxyethyl starch.

## Discussion

The pilot laboratory study revealed that urine caspase-cleaved cytokeratin-18 epitope M30 indicative of renal tubular cell apoptosis could be a potential early biomarker of AKI among patients without severe sepsis. These results, however, were not confirmed in the validation study including 197 consecutive patients with urine sample at ICU admission analyzed. In the pilot study, neither urine cell-free DNA nor HSP70 levels significantly differed between AKI and non-AKI patients regardless of the presence or absence of severe sepsis.

Apoptosis is considered as one of the key mechanisms contributing to ischemic AKI [5]. Generally, apoptosis starts to occur 6 to 12h from the insult [5]. To our knowledge, this is the first study to measure urine CK-18 M30 in the critically ill. Initial results show that urine levels of this epitope would work in quantifying apoptotic cell death in patients with bladder cancer [20]. As cytokeratin 18 is an abundant protein in the renal epithelium [8], and the detected plasma levels of this CK-18 M30 did not differ between AKI patients and their critically ill controls, the source of urinary CK-18 M30 is likely to be the renal epithelium.

In the pilot study, the urine levels of CK-18 M30 were significantly higher in non-septic AKI patients than in non-septic patients without AKI at 24 hours after ICU admission. At that time, no clinical features of AKI were observed and the urine output and creatinine levels did not differ between AKI and non-AKI patients, and CK-18 M30 seemed a promising novel biomarker. Therefore, we decided to study the urine CK-18 M30 levels at the time of ICU admission in a larger, consecutive, and unselected validation cohort. No significant differences in the urine CK-18 M30 epitope levels existed between AKI and non-AKI patients regardless of the sepsis or CKD status, however.

In the validation study we analyzed the urine samples obtained at the time of ICU admission. Over 50% of the patient developed AKI within the first 12h and very few later than 24h of ICU treatment. In the pilot study the median time from sample obtained at H24 (in which the significant difference was detected) to the development of AKI was 21 hours. Currently, no data regarding the evolution of CK-18 M30 levels in established AKI are available. However, we believe that it would be unlikely that patients who present with AKI within 12h of sampling would not have increased levels of CK-18 M30 if it would be a biomarker of developing AKI. Moreover, among non-septic AKI patients, the CK-18 M30 levels were significantly higher among those with AKI within the first 12h compared to those diagnosed later, but difference was not significant in comparison to non-AKI patients. Therefore, the different timing of samples analyzed in the validation study is unlikely to explain why the results of the pilot study could not be replicated. Possibly, the occurring apoptosis in the renal tubular cells may not convey such increased levels of CK-18 M30 levels in urine that would be detectable with reasonable sensitivity or there are multiple sources of CK-18 M30 measured in urine to confound the results.

Urine cell-free DNA levels have not been studied in the critically ill, but elevated levels of plasma cell-free DNA have been detected in critically ill patients with severe sepsis [21] and acute lung injury [22] indicating increased cell damage. In the current study, urine or plasma levels of cell-free DNA did not differ between non-AKI and AKI patients with or without sepsis, although apoptosis and other cell damage occurring in the renal tubular epithelium could theoretically lead to increased urinary levels of cell-free DNA. Possibly the timing of sampling in the pilot study was too early to detect differences in urine cell-free DNA between patients who developed AKI and those who did not.

Urine HSP70 levels have been measured in a small pilot study [14] and in a study consisting of AKI 17 patients and 20 controls diagnosed with the Acute Kidney Injury Network (AKIN) criteria and a further validation study with 10 AKI patients and 12 controls [15]. These studies have shown HSP70 to rise early before the clinical diagnosis of AKI [14, 15]. Our results from 30 AKI patients did not confirm the results of these previous reports. HSP has been reported to be a stable marker for a at least 9 months [23], and we do not believe that the longer time from sampling to analyses would explain why we did not detect difference between AKI and non-AKI patients in HSP70 levels. We detected very low plasma levels of HSP70 in all patient groups as a previous report among CKD patients [24]. Theoretically, higher levels of HSP70 could suggest presence of protective mechanisms to prevent apoptosis.

The strength of the pilot study is that we evaluated biomarker levels both in urine and plasma before the clinical diagnosis of AKI. In addition, we used the complete KDIGO classification to define AKI. Several limitations in our study, however, should be addressed. First, we could not assess the kinetics of these markers after the onset of AKI, as samples were obtained only at 0 and 24 h. Second, as the main interest of the study was urine levels of these markers, anuric AKI patients could not be included which decreased the number of stage 3 AKI patients. However, there were only 8 (4%) patients excluded from the validation cohort due to anuria at ICU admission. Third, patients with severe sepsis diagnosed on ICU admission who develop AKI only a few days later may differ from those severe sepsis patients who have AKI already when admitted. However, the 90-day mortality of septic AKI patients in this sub-study corroborated that of all septic AKI patients in the FINNAKI study [25] indicating that our sample in the pilot study was representative. Fourth, we did not normalize the urine biomarker concentrations to urine creatinine level, which might have amplified the signal in AKI. However, interpretation of urine creatinine–normalized biomarker levels may be cumbersome in the non-steady state conditions that occur in AKI [26]. Finally, as AKI is a multifactorial syndrome septic and especially non-septic AKI patients may have several potential insults to the kidney. Despite prospective detailed data in this study defining the main insult behind AKI reliably was not possible among non-septic AKI patients.

## Conclusions

Despite the promising results in the pilot study, urine caspase-cleaved cytokeratin-18 epitope M30 level measured at ICU admission was not significantly higher among patients developing AKI compared to non-AKI patients regardless of the presence of severe sepsis or CKD. Urine HSP70 or cell-free DNA levels did not differ between AKI and non-AKI patients. Our findings do not support that apoptosis detected with cytokeratin-18 epitope M30 level would be useful in assessing the development of AKI in the critically ill.

## Supporting Information

S1 Dataset Patient characteristics and laboratory values in the pilot study. (SAV)

S2 Dataset Patient characteristics and laboratory values in the validation study. (SAV)

S1 Table Characteristics of patients with and without acute kidney injury (AKI) in the pilot study. (PDF)

S2 Table Plasma biomarker levels in patients with and without acute kidney injury (AKI) in the pilot study. (PDF)

S3 Table Plasma biomarker levels in non-septic patients with and without acute kidney injury (AKI) in the pilot study. (PDF)

S4 Table Characteristics of patients with and without acute kidney injury (AKI) in the validation study. (PDF)
