# Supplementary material for: Urinary Biomarkers Indicative of Apoptosis and Acute Kidney Injury in the Critically Ill
Source: PLoS One. 2016 Feb 26;11(2):e0149956. doi: 10.1371/journal.pone.0149956 (PMC4769222; doi:10.1371/journal.pone.0149956)
Supplement: S1 Table — (PDF) [file pone.0149956.s003.pdf]

S1 Table. Characteristics of patients with and without acute kidney injury (AKI) in the pilot study.

| | AKI (n=30) | No AKI (n=30) | P-value |
|---|---|---|---|
| Age | 68 [50.5-76.5] | 65 [51.5-74.3] | 0.877 |
| Male sex | 19/30 (63.3) | 19/30 (63.3) | >0.999 |
| Measured baseline creatinine | 76.0 [58.0-79.5][a] | 80.0 [60.5-89.5] [a] | 0.361 |
| Hypertension | 14/29 (46.7) | 13/30 (43.3) | 0.796 |
| Diabetes mellitus | 5/30 (16.7) | 8/30 (26.7) | 0.532 |
| Number of received nephrotoxic agents prior to ICU admission[b] | 1.0 [0.0-2.0] | 1.0 [0.0-2.0] | 0.834 |
| Operative admission | 8/30 (26.7) | 14/30 (46.7) | 0.180 |
| Emergency admission | 28/30 (93.3) | 29/30 (96.7) | >0.999 |
| SAPS II score | 39.5 [31.8-50.0] | 37.5 [31.0-52.0] | 0.574 |
| Non-renal non-age SAPS II score | 25.5 [15.8-34.8] | 25.5 [16.8-33.8] | 0.988 |
| SOFA score, maximum | 8.5 [7.5-11.0] | 7.5 [6.8-10.0] | 0.088 |
| Ventilatory treatment | 27/30 (90.0) | 26/30 (86.7) | >0.999 |
| Vasoactives on day 1 | 27/30 (90.0) | 23/30 (76.7) | 0.299 |
| Severe sepsis | 10/30 (33.3) | 10/30 (33.3) | >0.999 |
| Length of ICU stay | 4.3 [2.5-7.0] | 2.8 [2.1-4.6] | 0.049 |
| Highest creatinine on admission day or within 48h prior ICU admission | 78.0 [60.5-91.0] | 69.5 [52.8-91.0] | 0.275 |
| Urine output, first 24h | 2345 [1661-3161] | 2336 [1849-3288] | 0.739 |
| Dead by day 90 | 9/30 (30.0) | 3/30 (10.0) | 0.104 |

Data expressed as median [IQR] or number/total number (%). ICU; intensive care unit, SAPS; Simplified Acute Physiology Score, SOFA; Sequential Organ Dysfunction Assessment

[a] Includes data from 17 of 30 subjects

[b] Included (maximum of 6): radiocontrast agent, aminoglycan or peptidoglycan antibiotics, angiotensin converting enzyme inhibitors or angiotensin receptor blockers, non-steroidal anti-inflammatory drugs, diuretics or hydroxyethyl starch.
