# Supplementary material for: Urinary Biomarkers Indicative of Apoptosis and Acute Kidney Injury in the Critically Ill
Source: PLoS One. 2016 Feb 26;11(2):e0149956. doi: 10.1371/journal.pone.0149956 (PMC4769222; doi:10.1371/journal.pone.0149956)
Supplement: S2 Table — (PDF) [file pone.0149956.s004.pdf]

S2 Table. Plasma biomarker levels in patients with and without acute kidney injury (AKI) in the pilot study.

	AKI (n=30)	No AKI (n=30)	P-value
**Caspase-cleaved cytokeratin-18 epitope M30 (U/L)**			
-0h	191.0 [154.5-279.3]	181.5 [139.5-302.5]	0.894
-24h	189.5 [153.5-278.8]	179.5 [136.0-238.0]	0.220
-highest	188.0 [161.5-302.3]	237.0 [177.3-390.5]	0.848
**Cell-free DNA (GE/mL)**			
-0h	16671 [6574-38231]	17160 [8659-43077]	0.976
-24h	15557 [7800-31039]	15671 [9629-22485]	0.859
-highest	15035 [7660-33218]	21196 [9479-40165]	0.848
**HSP (ng/mL)**			
-0h	0.47 [0.00-1.33]	0.00 [0.00-0.75]	0.255
-24h	0.00 [0.00-0.63]	0.00 [0.00-0.60]	0.947
-highest	0.47 [0.00-1.52]	0.20 [0.00-0.80]	0.549

Data expressed as median [IQR].
