# Supplementary material for: Urinary Biomarkers Indicative of Apoptosis and Acute Kidney Injury in the Critically Ill
Source: PLoS One. 2016 Feb 26;11(2):e0149956. doi: 10.1371/journal.pone.0149956 (PMC4769222; doi:10.1371/journal.pone.0149956)
Supplement: S3 Table — (PDF) [file pone.0149956.s005.pdf]

S3 Table. Plasma biomarker levels in non-septic patients with and without acute kidney injury (AKI) in the pilot study.

	AKI (n=20)	No AKI (n=20)	P-value
**Caspase-cleaved cytokeratin-18 epitope M30 (U/L)**			
-0h	168.0 [151.5-251.5]	181.5 [140.3-303.5]	0.620
-24h	185.0 [148.5-239.8]	192.0 [136.0-238.0]	0.862
-highest	188.0 [161.5-302.3]	237.0 [177.3-390.5]	0.327
**Cell-free DNA (GE/mL)**			
-0h	12767 [4880-33217.5]	14983 [9281-39637]	0.620
-24h	11168 [7050-20809]	17121 [6260-23125]	0.620
-highest	15035 [7660-33218]	21296 [9479-40165]	0.461
**HSP (ng/mL)**			
-0h	0.47 [0.00-1.52]	0.00 [0.00-0.73]	0.183
-24h	0.00 [0.00-0.47]	0.00 [0.00-0.45]	0.862
-highest	0.47 [0.00-1.52]	0.20 [0.00-0.80]	0.398

Data expressed as median [IQR].
