# Supplementary material for: Urinary Biomarkers Indicative of Apoptosis and Acute Kidney Injury in the Critically Ill
Source: PLoS One. 2016 Feb 26;11(2):e0149956. doi: 10.1371/journal.pone.0149956 (PMC4769222; doi:10.1371/journal.pone.0149956)
Supplement: S4 Table — (PDF) [file pone.0149956.s006.pdf]

S4 Table. Characteristics of patients with and without acute kidney injury (AKI) in the validation study.

	AKI (n=79)	Non-AKI (n=118)	P-value
Age	66.0 [55.0-76.0]	62.5 [49.8-72.0]	0.128
Male sex	52/79 (65.8%)	78/118 (66.1%)	>0.999
Hypertension	42/78 (53.8%)	54/116 (46.6%)	0.380
Diabetes mellitus	21/79 (26.6%)	18/118 (15.3%)	0.068
Chronic kidney disease	14/79 (17.7%)	4/117 (3.4%)	0.001
Number of received nephrotoxic agents prior to ICU admission[a]	2 [1-2]	1 [0-2]	0.005
Operative admission	28/79 (35.4%)	34/118 (28.8%)	0.327
Emergency admission	68/74 (91.9%)	112/117 (95.7%)	0.342
SAPS II score	40.0 [33.0-44.0]	34.5 [27.0-44-0]	0.01
Non-renal non-age SAPS II score	21.0 [15.0-28.0]	23.0 [13.0-29.8]	0.748
SOFA score, maximum	9 [7-12]	6 [4-9]	<0.001
Ventilatory treatment	53/79 (67.1%)	70/118 (59.3%)	0.296
Vasoactives d1	57/79 (72.2%)	51/118 (43.2%)	<0.001
Urine output, first 24h	1365 [820-2357]	2760 [1813-3805]	<0.001
Severe sepsis	31/79 (39.4%)	26/118 (22.0%)	0.011
Length of ICU stay	3.9 [2.4-7.2]	2.2 [1.1-4.7]	<0.001
Dead by day 90	22/79 (27.8%)	22/118 (18.6%)	0.163

Data expressed as median [IQR] or number/total number (%).ICU; intensive care unit, SAPS; Simplified Acute Physiology Score, SOFA; Sequential Organ Dysfunction Assessment
[a] Included (maximum of 6): radiocontrast agent, aminoglycan or peptidoglycan antibiotics, angiotensin converting enzyme inhibitors or angiotensin receptor blockers, non-steroidal anti-inflammatory drugs, diuretics or hydroxyethyl starch.
